# Measles outbreak investigation in Artuma Fursi Woreda, Oromia Zone, Amhara Region, Ethiopia, 2018: a case control study

**DOI:** 10.1186/s13104-019-4806-y

**Published:** 2019-11-21

**Authors:** Mengistie Kassahun Tariku, Sewnet Wongiel Misikir

**Affiliations:** 1 Aneded Woreda Health Office, East Gojjam, Amhara Region Ethiopia; 2 Felege Hiote Specialized Referral Hospital, Bahir Dar, Ethiopia

**Keywords:** Measles, Outbreak, Artuma Fursi Woreda

## Abstract

**Objective:**

To confirm the existence of Outbreak, describe cases in person, place and time, and identify determinants of the outbreak. Unmatched case control study in the ratio of 1:4 (38 cases and 152 controls) was conducted in Artuma Fursi Woreda from July 13 to August 1/2018. Data were collected with standard questionnaires. Collected data were entered into Epi Info version 7 and exported to Statistical package for social science (SPSS) version 23 for analysis.

**Results:**

A total of 38 cases and 1 death with attack rate and case fatality rate 11.8/100,000 and 2.6%, respectively. All study participants had not vaccination history. Females and age group 5–14 were more affected. Being 5–14 years old versus (vs) ≥ 15 years [adjusted odd ratio (AOR) = 3.53; 95% CI 1.52–8.45)], contact with cases vs no contact with cases [AOR = 2.78; 95% CI 1.23–8.67] and travel history 7–18 days prior onset of illness vs no travel history [AOR = 2.53; 95% CI 1.31–7.24] were significantly associated with contracting measles. Routine and supplement immunization should be strengthened to reduce future occurrence of outbreak.

## Introduction

Measles is a highly contagious, serious disease caused by a virus [1, 2]. Any person with fever, and non-vesicular rash, and cough, runny nose or red eyes is the suspect of measles case. Confirmed measles case is a suspected case with laboratory confirmation (positive for immunoglobulin M (IgM) antibody) [3]. Measles is caused by a virus in the paramyxovirus family and it is normally passed through direct contact and through the air. The virus infects the respiratory tract, and then spreads throughout the body. Measles is a human disease and is not known to occur in animals [4].

Before the introduction of measles vaccine in 1963 and widespread vaccination, major epidemics occurred approximately every 2–3 years and measles caused an estimated 2.6 million deaths each year [1, 2].

The disease remains one of the leading causes of death among young children globally, despite the availability of a safe and effective vaccine [1, 5]. Approximately 89 780 people died from measles in 2016—mostly children under the age of 5 years [1].

In populations with high levels of malnutrition, particularly vitamin A deficiency, and a lack of adequate health care, about 3–6%, of measles cases result in death, and in displaced groups, up to 30% of cases result in death. Women infected while pregnant are also at risk of severe complications and the pregnancy may end in miscarriage or preterm delivery. People who recover from measles are immune for the rest of their lives [4, 6].

The case fatality rate of measles disease was increased with travel distance from the nearest health facility. The difference in the access to health care can affect the burden of the disease in low-income settings [7].

In countries with weakly functioning health care systems, measles mortality reduction or elimination was addressed through second dose periodic supplementary immunization activities (SIAs) in the form of mass campaigns. Strengthen routine vaccination service can improve measles control, measles death reduction and elimination program which can help build up primary health care capacity [8].

Measles has been difficult to eliminate historically, but there is a global goal to eradicate it, and measles was first eliminated in the Americas [9].

In Ethiopia, implementation of measles death reduction approach was launched in 2002. The national Expanded Program on Immunization was started in 1980, and the first dose of measles-containing vaccine (MCV1) is given at or after the ninth month of age [10]. About 54% of 12–23 months children were received routine measles vaccination in 2016 [11]. Supplementary immunization activities were conducted every 2 to 4 years, targeting all children between 9 months and 14 years of age [12].

Suspected measles outbreak is defined as occurrence of five or more reported suspected cases in 1 month in a defined geographic area, like, Kebele (the smallest administrative unit of the Woreda in Ethiopia), district or health facility catchment area whereas Confirmed measles outbreak is defined as occurrence of three or more laboratory confirmed cases in 1 month in a defined geographic area, like, Kebele, district or health facility catchment area [3].

Measles outbreaks can occur in areas with high vaccination coverage [13]. It is also common in many low- income countries but there is limited information in confirming the outbreak, describing the outbreak in terms of person, please and time, and identifying determinants of outbreak [14].

Measles is one of immediately reportable disease in Ethiopia. On Wednesday morning, July 12/2018, one Suspected measles case was reported from Golbo Arba Kebele in Artuma Fursi Woreda. Within 2 h, three field Epidemiology residents and one Artuma Fursi Woreda health office Public health Emergency officer visited the reported Kebele to confirm the existence of the outbreak, describe cases in person, place and time, and identify determinants of measles case status.

## Main text

### Methods

#### Study setting and period

Unmatched case–control study design was done from July 13-Agust 1/2018 in Golbo Arba Kebele, Artuma Fursi Woreda, Oromia Zone, Amhara Region, Ethiopia. The Woreda is located about 305 km from Addis Ababa (the capital city of Ethiopia) and 575 km from Bahir Dar. The area of the Woreda is 782.22 km^2^ with the total population 103,611. The Woreda is administratively divided into 25 kebeles. There are 6 health centers and 25 health post in the Woreda. Golbo Arba Kebele has total population of 3229 [15].

### Sampling size and sampling technique

The sample size was calculated in Epi Info version 7. Our sample size was designed to determine significance of the relationship between vaccination and case status. Based on an alpha value of 0.05, and a predicted prevalence of non-vaccination of 31% among controls and 86.2% among cases [16], we would need 38 cases enrolled in the study. However, due to missing data with the vaccination variable, we did not include it in the final multivariable analysis. We ended up enrolling all measles cases in the area in the study period, and sampled cases to controls based on the 1:4 ratio to increase efficiency of results. All eligible cases [any person with fever and maculopapular (non-vesicular) generalized rash and cough, Coryza or conjunctivitis (red eyes)] were included in the study whereas all eligible controls (neighbors or family member of cases who did not have fever, non-vesicular generalized rash and cough) were selected by lottery method simple random sampling. Both cases and controls who had history of measles in the previous years were excluded.

#### Measurement

The data were collected from house-to-house search for measles case. A standard questionnaire was used to collect data using face to face interview technique by 3 Field Epidemiology residents. Serum specimens were collected from five suspected measles cases and sent to the national measles laboratory for IgM test and a test done as per the global and national guidelines.

#### Data analysis

Data were coded and entered using Epi-Info 7 and analyzed by SPSS version 23. Data cleaning and recoding was preformed, then descriptive, bivariate and multivariable binary logistic regression was done. Bivariate analysis was computed. Each variable that has *p*-value ≤ 0.25 was entered into multivariable analysis. In multivariable analysis, every variable that has p-value < 0.05 was considered significance. Results were presented using text, tables and graphs.

### Result

#### Descriptive epidemiology

A total of 38 measles cases, 1 death and 152 controls and that fulfill standard case definition were identified. The median age of the cases was 7.5 years [interquartile range (IQR) = 9.25 years] and for that of the controls was 25 years [IQR = 17 years]. In this investigation, higher attack rate [(AR) 139.4/10,000] was reported among females. Case fatality rate (4.3%) was also higher among females. Higher AR (291.0/10,000) was reported among age group 5–14 years. Case fatality rate (4%) was also higher among age group 5–14 years.

More than half, 23 (57.1%), of cases and 81 (53.3%), of controls were female. Regarding to educational status of mothers/care givers, 27 (71.1%) of mothers/care givers of cases and 102 (67.1%) of mothers/care givers of controls were unable to read and write (Table 1).

**Table 1:** Socio demographic characteristics of Study participant in Artuma Fursi Woreda, Oromia Zone, Northeast Ethiopia, 2018

Variables	Respondent status	
	Cases (n = 38), N (%)	Controls (n = 152), N (%)
Sex		
Female	23 (57.1)	81 (53.3)
Male	15 (42.9)	71 (46.7)
Age in year		
<1	2 (5.3)	8 (5.3)
1–4	10 (26.3)	12 (7.9)
5–14	24 (63.2)	53 (34.9)
≥ 15	2 (5.3)	79 (52.0)
Educational status of study participants		
Not eligible	12 (31.6)	20 (13.2)
Unable to read and write	19 (50.0)	52 (34.2)
Read and write but have no formal education	5 (13.1)	27 (17.8)
Primary	2 (5.3)	53 (34.9)
Educational status of mothers/care givers		
Unable to read and write	27 (71.1)	102 (67.1)
Read and write but have no formal education	8 (21.0)	32 (21.1)
Primary	3 (7.9)	18 (11.8)
Do measles have medical treatment		
No	27 (71.1)	91 (59.9)
Yes	11 (28.9)	61 (40.1)
Contact with case		
No	32 (84.2)	111 (73.0)
Yes	6 (15.8)	41 (27.0)
History travel 7–18 days prior onset of illness		
Yes	15 (39.5)	36 (23.7)
No	23 (60.5)	116 (76.3)

Distribution of cases by place: In this outbreak, all measles cases were from one gote.

**Fig. 1:**
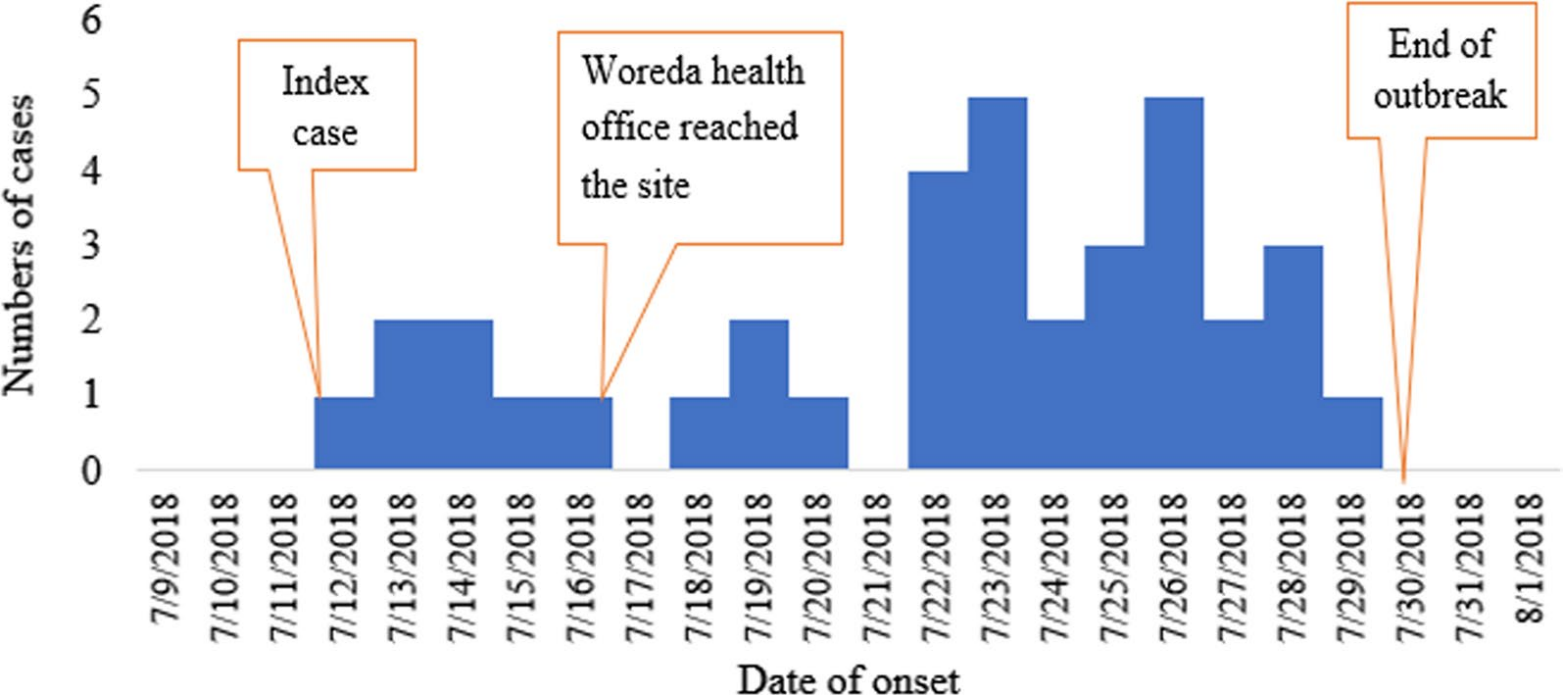
Epi curve shows distribution measles cases by date of onset of sign and symptom in Artuma Fursi Woreda, Oromia Zone, Northeast Ethiopia, 2018

Distribution cases by Time: The index case showed sign and symptom on 7/12/2018. The index case had travel history to chefa Robite and had history of contact with person who came from Saudarbia who had similar sign and symptom. More than one-fourth, 10 (26.3%) of cases had onset of symptom on July 23/2018 and July 26/2018 (Fig. 1).

Laboratory investigation: Among the total samples, 3 (60%) of samples were IgM positive for measles. Other remaining cases were confirmed by epidemiological linkage.

#### Analytical study

In Bivariate analysis, age of study participants, educational status of mothers/care givers, have or have not medical treatment for measles, contact history with cases and travel history 7–18 days prior onset of illness had p-value less than or equal to 0.25 and these variables were included in multivariable analysis (Table 2).

**Table 2:** Multivariable analysis of factors associated with measles outbreak in Artuma Fursi Woreda, Oromia Zone, Amhara region, Ethiopia, 2018

Variables	Respondent status		COR (95% CI)	AOR (95% CI)	p-value
	Cases = 38	Controls = 152			
Age in year					
<1	2	8	9.9 (1.01–15.21)	6.42 (2.12–10.03)	0.001
1–4	10	12	32.07 (1.34–46.84)	3.13 (1.08–6.41)	< 0.0001
5–14	24	53	17.89 (8.29–27.31)	3.53 (1.52–8.45)	0.002
≥15	2	79	1	1	0.001
Educational status of mothers/care givers					
Unable to read and write	27	98	1.29 (1.01–6.68)	1.90 (0.85–4.23)	0.091
Read and write but has no formal education	8	34	1.57 (1.14–7.78)	1.4 (0.50–5.11)	0.054
Primary	3	20	1	1	0.070
Do measles have medical treatment					
No	27	91	1.65 (0.72–3.63)	1.45 (0.63–4.23)	0.063
Yes	11	61	1	1	
Contact with case					
Yes	6	41	0.51 (0.18–3.98)	2.78 (1.23–8.67)	< 0.0001
No	32	111	1	1	
History travel 7–18 days prior onset of illness					
Yes	15	36	2.10 (1.22–5.71)	2.53 (1.31–7.24)	< 0.0001
No	23	116	1	1	

In Multivariable analysis, age of study participants, contact history with cases (AOR = 2.78, 95% CI 1.23–8.67) and travel history 7–18 days prior onset of illness (AOR = 2.53, 95% CI 1.31–7.24) were significantly associated with measles (Table 2).

### Discussion

In this outbreak investigation, the overall attack rate was 11.8/100,000. This is higher than the study conducted in Somalia region 28.6/100,000 [8], and the investigation conducted in Guji Zone, Oromia region, Ethiopia [17]. This difference might be due to vaccination coverage, 14.8% and 16% of measles cases had history of vaccination in Somalia region and Guji Zone, respectively but in this investigation 100% of measles cases had not vaccination history.

The overall attack of this outbreak investigation is consistent with the investigation conducted in Pakistan which had the overall attack rate 11.3/100,000 [18]. This might be due to vaccination status similarity of measles cases because all cases in Pakistan had no vaccination history.

In this study, about 57.1% (95% CI, 42.9–71.4%) of the cases were females. This is consistent with investigation conducted in Somalia 45.3% [8] and Pakistan 51% [18]. This might be due to females are more vulnerable for malnutrition [4, 6] because of cultural influence.

In this outbreak investigation, the age specific attack rate (29.1/100,000) is higher in the age of 5–14 years. This is discrepancy with the study conducted in Somali region Ethiopia that showed that the more affected age group were < 1 year [17]. This might be due to the age group 5–14 years of children had immunization in routine or supplemental immunization activity (SIA) in Somali region. In this study, all age group of the cases had no history of immunization.

The Cumulative CFR rate of this investigation was 2.6% (95% CI, 2.5–8.6%). This is consistent with the studies conducted in Pakistan 7.27% [6] and Nigeria 3.9% [19]. But this finding is higher than the study conducted in Somalia region (Ethiopia) 1.2% [8] and Guji Zone, Oromia region (Ethiopia) 0.2% [17]. This might be due to late outbreak response because the Woreda Health Office gave response after the index case dead.

In this investigation, age is significantly associated with contracting measles. Being age between 5 and 14 years were almost 3.5 times more likely contracting measles than peoples who is age ≥ 5 years. This is consistent with the investigation conducted in Nigeria which showed that case and death of measles were significantly associated with age [19].

Persons who had contact with measles case were almost 3 times more likely in contracting measles than persons who had not contact with measles case. This is consistent with the study conducted in Zimbabwe [20].

In this outbreak investigation, persons who had history of travel 7–18 days prior onset of illness were almost 2.5 times more likely in contracting measles in the study area than those who had not travel history 7–18 days prior to the onset of illness. This is in line with the study conducted in china [21]. The justification of this result might be traveled persons might prone to contracting measles.

### Conclusion

Measles outbreak was occurred at Golbo Arba Keble in Artuma Fursi Woreda revealed that all measles cases not immunized. Age group of 5–14 years had higher AR. All cases were from one gote. Age, contact with cases and having history of travel 7–18 days prior onset of illness were factors for contracting measles It is recommended that early and proper response should be given. People who have travel history should be traced, and community surveillance and outreach vaccination should be strengthened.

## Limitation

The immune status of the controls was not measured. This may lead selection bias. Due to cultural influence, some mothers/care givers might hide some measles cases. Case definition was not laboratory confirmation. Thus, some measle cases might be missed classified.

## Data Availability

The data sets generated during the current study are available from corresponding author on reasonable request.

## Abbreviations

AOR: adjusted odd ratio
AR: attack rate
CFR: case fatality rate
CI: confidence interval
COR: crude odd ratio
